# Stress myocardial perfusion cardiac magnetic resonance imaging vs. coronary CT angiography in the diagnostic work-up of patients with stable chest pain: comparative effectiveness and costs

**DOI:** 10.1186/1532-429X-15-S1-O9

**Published:** 2013-01-30

**Authors:** Tessa S Genders, Steffen E Petersen, Francesca Pugliese, Amardeep Ghosh Dastidar, Kirsten E Fleischmann, Koen Nieman, Myriam Hunink

**Affiliations:** 1 Centre for Advanced Cardiovascular Imaging, Barts and The London School of Medicine and Dentistry, London, UK; 2 Department of Epidemiology, Erasmus University Medical Centre, Rotterdam, the Netherlands; 3 Division of Cardiology, UCSF Medical Center, San Francisco, CA, USA; 4 Department of Cardiology, Erasmus University Medical Centre, Rotterdam, the Netherlands

## Background

To determine the comparative effectiveness and costs of coronary CT angiography (CCTA) and stress cardiac magnetic resonance imaging (CMR) for diagnosing coronary artery disease (CAD).

## Methods

A Markov micro-simulation model for 60-year-old patients with stable chest pain was developed, analyzing the perspective of the United States (US), United Kingdom (UK), and the Netherlands (NL).

CCTA, CMR, and CCTA+CMR (CCTA, if positive followed by CMR) were considered and compared to direct catheter-based angiography (CAG) and no testing. The strategies were considered both as conservative strategy (patients with mildly-positive test results are not referred for CAG), and as invasive strategy (all patients with positive test results are referred for CAG). Outcome measures included lifetime costs, quality-adjusted life years (QALY), and radiation exposure.

## Results

Differences in effectiveness (QALYs) across diagnostic strategies were very small (range 0.001-0.016). For 60-year old men and women with a pre-test probability of 30% (and up to 70-90%, depending on the country considered), the CCTA, CMR, and CAG strategies were dominated, because the CCTA+CMR-conservative strategy was slightly more effective, and less expensive. Compared to the CCTA+CMR-conservative strategy, the CCTA+CMR-invasive strategy was slightly more costly and slightly more effective. The CCTA+CMR-invasive strategy was cost-effective for the US and NL, but not for the UK. When patients with false-negative test results were assumed to remain false-negative for 3 years, differences between strategies increased, and the CCTA-invasive strategy became cost-effective for UK and NL.

## Conclusions

Quality-adjusted life expectancy was similar across strategies. The CCTA+CMR strategy was cost-effective up to a pre-test probability of 70-90%, depending on the country. Above these thresholds, the CMR-strategy was cost-effective.

## Funding

This research was supported by a Health Care Efficiency grant from the Erasmus University Medical Centre, Rotterdam. S.E.P. and F.P. were directly funded by the Barts and the London National Institute for Health Research Cardiovascular Biomedical Research Unit. A.G.D. was directly funded through the Barts and The London Charity (437/1412). K.E.F. was directly funded through a grant from NIH (NIH-NHLBI award no. R21HL112255).

